# Moving toward the Inclusion of Epigenomics in Bacterial Genome Evolution: Perspectives and Challenges

**DOI:** 10.3390/ijms25084425

**Published:** 2024-04-17

**Authors:** Iacopo Passeri, Francesca Vaccaro, Alessio Mengoni, Camilla Fagorzi

**Affiliations:** Department of Biology, University of Florence, 50121 Firenze, Italy; iacopo.passeri@unifi.it (I.P.); francesca.vaccaro@unifi.it (F.V.); camilla.fagorzi@unifi.it (C.F.)

**Keywords:** prokaryotic DNA methylation, bacterial epigenomics, bacterial genome evolution

## Abstract

The universality of DNA methylation as an epigenetic regulatory mechanism belongs to all biological kingdoms. However, while eukaryotic systems have been the primary focus of DNA methylation studies, the molecular mechanisms in prokaryotes are less known. Nevertheless, DNA methylation in prokaryotes plays a pivotal role in many cellular processes such as defense systems against exogenous DNA, cell cycle dynamics, and gene expression, including virulence. Thanks to single-molecule DNA sequencing technologies, genome-wide identification of methylated DNA is becoming feasible on a large scale, providing the possibility to investigate more deeply the presence, variability, and roles of DNA methylation. Here, we present an overview of the multifaceted roles of DNA methylation in prokaryotes and suggest research directions and tools which can enable us to better understand the contribution of DNA methylation to prokaryotic genome evolution and adaptation. In particular, we emphasize the need to understand the presence and role of transgenerational inheritance, as well as the impact of epigenomic signatures on adaptation and genome evolution. Research directions and the importance of novel computational tools are underlined.

## 1. Introduction

Epigenomic modifications, in particular DNA methylation, have been found to be involved in several mechanisms, including the control of gene expression, cellular differentiation, and DNA repair. In bacteria, DNA methylation has been described in detail in relation to cell cycle progression, self-DNA recognition (restriction–modification systems), DNA repair, and more recently, DNA–protein interactions and gene expression control [1,2,3]. Thanks to genome sequencing technologies, enabling the identification of methylated bases at the genome level, studies on epigenetic modification in eukaryotes and in bacteria have gained momentum. Investigations have found that genome-wide DNA methylation patterns can be highly variable even inside the same bacterial species and may affect adaptive traits, such as phenotypic variation, and gene transfer among closely related strains [1,2].

In this review, we aim to summarize the main roles that DNA methylation has in bacteria, focusing on the prevailing lack of knowledge about its importance in gene expression control and the need for tools to extract and process the information from genome-wide DNA methylation data to gain novel insights into the importance of epigenomic modification in bacterial behavior.

After introducing the mechanism of action of DNA methyltransferases, the enzymes introducing methyl groups into DNA bases, we present evidence of the involvement of DNA methylation in core processes and gene expression regulation. We then discuss the possible involvement of DNA methylation in the genome evolution of bacteria and the current limits of computational tools for methylation data analysis, suggesting future research directions for understanding bacterial epigenomics.

## 2. Classification and Mode of Action of DNA Methyltransferases

DNA methyltransferases (DNA MTases) are the enzymes responsible for catalyzing the transfer of a methyl group from the donor S-adenosyl-l-methionine (SAM) to the target DNA [4]. DNA MTases have three main catalytic domains and the target recognition domain (TRD) is the most varied across biological kingdoms, which may reflect evolutionary diversification from a common ancestor [5]. There are three known forms of methylation: C-5 and N-4 cytosine methylation (5mC, 4mC) and N-6 adenine methylation (6mA). The classification of DNA MTases is based on sequence homology, cofactor usage, and target recognition. The primary classes are maintenance (type I and type II) and de novo (type III) DNA MTases. Although all of these enzymes are frequently associated with restriction–modification (R-M) systems, they can also function independently as orphan DNA MTases, without a matched restriction enzyme (REase) partner, contributing to processes such as cell regulation, cell replication, DNA repair, and population evolution [6]. Type II DNA MTases are R-M systems and represent the most prevalent type in prokaryotes. Unlike the multicomplex structure of type I and type III DNA MTases, type II can act as a single protein on short palindromic recognition sequences. They methylate adenine or cytosine on both strands and are crucial for regulating gene expression. In bacteria, three categories of type II DNA MTases have been recognized based on the specific nucleotide base modified by the transfer of a methyl group: N-6-methyladenine (class I), N-4-methylcytosine (class II), or C-5-methylcytosine (class III). Frequently, these DNA MTases are linked to a corresponding REase, forming a R-M system that shields bacterial cells against foreign DNA intrusion. When DNA MTases are uncoupled from REase, they are referred to as orphan DNA MTases. Such orphan DNA MTases can be involved in regulatory functions. Similar to type I, type III DNA MTases act as a complex and have separate methyltransferase and restriction endonuclease activities. They recognize asymmetric sequences, methylating adenine on one strand. The catalytic activity of DNA MTases involves the initial recognition and binding of the enzyme to its specific target sequence, characterized by palindromic or asymmetric motifs. Upon binding, the DNA MTase induces a conformational change, leading to the extrusion of the target base from the DNA helix. This dynamic process, known as “base flipping”, exposes the target base for methylation while maintaining the overall structural integrity of the DNA. The methyl group can be added to the cytosine C-5 (m5C) by exocyclic DNA MTases, or to cytosine N-4 (m4C) and adenine N-6 (m6A) by endocyclic DNA MTases. Some features of type I and type II methyltransferases can be found in a fourth class of enzymes, type IV MTases. The common feature of these enzymes is that m6A-specific methyltransferase and restriction endonuclease activities are combined in one polypeptide chain. Furthermore, the REase activity is positively affected by SAM, but ATP has no influence on the activity of the enzymes. Type IV enzymes usually recognize asymmetrical DNA sequences and cleavage occurs at a defined distance from the recognition site [7,8].

## 3. DNA Methylation Pattern Is Involved in Core Cellular Processes

Unlike eukaryotic DNA MTases, bacterial DNA methylation often exhibits specific motifs as targets for regulatory and functional roles. Paradigmatic examples of DNA methylation motifs in prokaryotes are found within R-M systems [9]. These systems, widespread in bacteria and archaea, serve as defense mechanisms against foreign DNA, such as bacteriophages, and for this reason, they are commonly considered a DNA-based immune system. In turn, bacteriophages employ diverse mechanisms to evade or exploit these methyl-dependent defenses, leading to a constant cycle of adaptation and counter-adaptation. Indeed, DNA methylation plays a pivotal role in distinguishing self- from non-self-DNA. The host genome is methylated at specific recognition sites, protecting it from cleavage by the related restriction enzymes (i.e., restriction endonuclease activity, which degrades foreign DNA by introducing double-strand breaks). In contrast, invading phage DNA, lacking the appropriate methylation pattern, becomes a target for restriction enzyme cleavage, thus allowing the infected cell to stop the infection [9].

The other paradigmatic examples of DNA methylation patterns are those related to cell cycle control and DNA repair systems. Here, the methylation status of the parental DNA strand is copied onto the newly synthesized strand during DNA replication. The accurate recognition and methylation of specific residues ensure the preservation of epigenetic marks in the daughter cell’s genome. This process, often referred to as ‘semi-conservative’ methylation, forms the basis of the continuity of epigenetic information in prokaryotic lineages. The Dam, Dcm, EcoKI, and CcrM DNA MTases provide the classical systems explaining the role of DNA methylation patterns in cell cycle control and DNA repair and the inheritance of DNA methylation patterns. The Dam methylase of Gammaproteobacteria, which belong to the class of type II DNA methylases, recognizes the GATC sequence and methylates the adenine residue within it. Dcm MTase, also a type II DNA MTase identified in Gammaproteobacteria, adds a methyl group to the second cytosine in the DNA sequence CCATGG. This enzyme plays roles in various cellular processes, including DNA repair, the regulation of gene expression, and protection against foreign DNA [10]. In certain strains of *E. coli*, the DNA methyltransferase EcoKI, a type I restriction–modification enzyme that methylates the adenine residues in the specific DNA sequences AAC(N6)GTGC and GCAC(N6)GTT, has been well described for its importance in protecting the bacterial DNA from being cleaved by the restriction enzyme EcoKI (HsdR), which recognizes the same DNA sequence but cuts unmethylated DNA [11]. In Alphaproteobacteria, most of the actual knowledge on DNA MTases is focused on CcrM, a type IV MTase which methylates the adenine residue of the motif GANTC [12,13]. This methylation mark is crucial during the early stages of DNA replication; as the DNA is copied, one of the daughter strands (the template strand) inherits the methylated adenine, while the other (the newly synthesized strand) is unmethylated. This transient asymmetry in methylation serves as a temporal signal, influencing processes such as DNA repair, recombination, and cell cycle progression [14]. The activity of CcrM in Alphaproteobacteria is also relevant for its cell cycle regulatory function; in fact, chromosome replication starts through the interaction of the replication initiation protein DnaA with the origin of replication, a process triggered by the full methylation of GANTC [15,16,17,18,19]. The methylation status of both DNA strands at GANTC sites also has implications in the bacterial differentiation processes taking place in endosymbiotic bacteroids in plant nitrogen-fixing symbiotic rhizobia. Here, the analysis of the genome distribution of methylated GANTC sites along the three main replicons of the rhizobial species *Sinorhizobium meliloti* provided evidence that CcrM activity is affected during bacteroid differentiation and could be a driving factor for genome endoreduplication of such terminally differentiated cells [15]. DNA repair and mismatch repair (MMR) systems work in tandem with cell cycle control methylation patterning, contributing to the accuracy of DNA methylation inheritance. The specificity of MMR is mainly for base–base mismatches and insertion/deletion mispairs generated during DNA replication and recombination [20]. During DNA repair, the type II restriction endonuclease MutH recognizes hemi-methylated DNA sites containing mismatches and removes the non-methylated DNA strand, ensuring that the methylated parental strand will be used as the template for repair-associated DNA synthesis [6,21]. This interplay between DNA methylation and repair mechanisms highlights the intricate balance required for maintaining epigenetic information during the continuous process of cellular division and indicates some of the fundamental roles of DNA MTases and DNA methylation patterns in prokaryotic cells’ core processes.

## 4. Impact of DNA Methylation Patterns on Gene Expression

**Figure 1:**
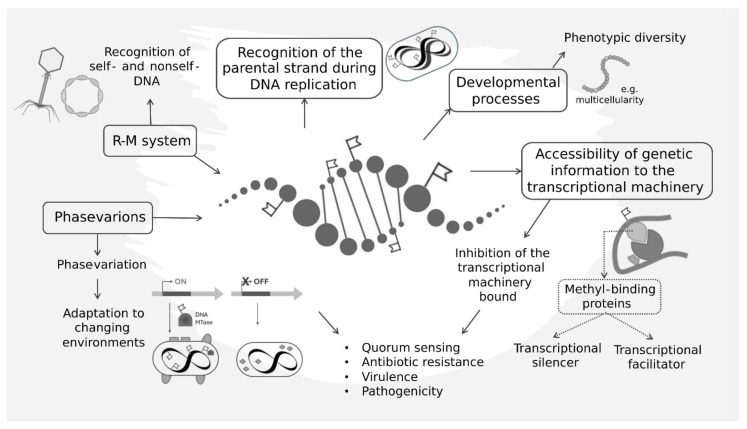
Effects of DNA methylation on cellular functions, adaptation, and evolution. DNA methylation plays a pivotal role in the replication of DNA, marking the parental strand to prevent and allow for the correction of replication errors. DNA methylation plays different roles in the adaptation of prokaryotes to the environment (e.g., quorum sensing, antibiotic resistance, virulence and pathogenicity, and developmental processes), modeling the accessibility of genetic material to the transcriptional machinery and thus influencing the transcription of genes. R-M systems, as well as the presence of phasevarions, shape the methylation pattern of prokaryotic cells through the expression of DNA MTases, with effects on environmental adaptation and the exchange of genetic material.

Prokaryotes exhibit a remarkable ability to sense and adapt to their environment. As in eukaryotes, DNA methylation could serve as a molecular memory that allows bacteria to ‘remember’ past environmental exposures and adapt their gene expression profiles accordingly [22]. In fact, environmental fluctuations brought to the need of modifying gene expression and this can involve epigenetic information; environmental stimuli, stressors, and growth conditions have been reported to modulate bacterial DNA methylation patterns [23]. Methylation patterns serve as molecular flags, dictating the accessibility of genetic information to the transcriptional machinery. Methylated DNA can impede the binding of RNA polymerases and/or accessory factors to promoter and control regions, thus hindering the initiation of transcription. This inhibition is observed in the regulation of virulence genes in certain pathogenic bacteria, where DNA methylation serves as a critical checkpoint to prevent the unwarranted expression of virulence factors in non-pathogenic conditions [21]. Additionally, DNA methylation-induced gene repression may involve the recruitment of methyl-binding proteins that act as transcriptional silencers [24]. These proteins recognize methylated DNA motifs and orchestrate the compaction of chromatin, rendering it inaccessible for transcriptional machinery [25]. Alternatively, DNA methylation can act as a facilitator by recruiting transcription factors to promote gene expression. Methylated cytosines within specific promoter regions create a binding platform for regulatory proteins, leading to enhanced recruitment of RNA polymerase. This facilitative effect is exemplified in the regulation of stress response genes in bacteria, where DNA methylation primes the transcriptional machinery for rapid activation in response to environmental challenges [26]. Stressors, nutrient availability, and other environmental cues influence the expression of DNA methyltransferases, leading to dynamic changes in methylation landscapes (Figure 1).

Examples of the effects of DNA methylation on bacterial gene expression profiles are accumulating. For example, a cytosine methylation-deficient mutant of *Escherichia coli* showed increased expression of the stress response sigma factor RpoS [26]. Adenine methylation changes have the potential to contribute to phenotypic adaptation in *Serratia marcescens* [27]. Furthermore, the difference in global DNA methylation patterns of cyanobacteria between unstressed and nutrient-stressed conditions supports the evidence of partially inheritable DNA methylation pattern changes [28]. Another example of bacteria’s ability to adapt to different environments by altering DNA methylation patterns is provided by *Klebsiella pneumoniae* in altered gravity conditions [29]. Here, the bacteria regulate gene expression patterns through DNA methylation and changes in genome structure, thus resulting in new phenotypes [29]. Moreover, different patterns of DNA methyltransferase activity in lineages of *Mycobacterium tuberculosis* have been found to be associated with preferences for distinct host environments (characterized by different hypoxia levels) and different disease courses in humans [30]. In addition, in bacterial colonies, phenotypic diversity is often observed, giving rise to subpopulations with distinct characteristics [31]. DNA methylation patterns contribute to this diversity by influencing the expression of genes involved in colony morphology. Still concerning differentiation processes, DNA methylation has been implicated in orchestrating developmental events in certain cyanobacteria, where a form of multicellularity and differentiation has been observed. Specific genes involved in cell differentiation and pattern formation are regulated by DNA methylation, influencing the formation of specialized cell types within the multicellular structure [32], as already indicated for CcrM in *Caulobacter crescentus* and rhizobia.

Several clues for the generalized importance of DNA methylation patterns in bacterial phenotypic changes stem from from the study of pathogenicity. Some examples include *Vibrio cholerae*, where methylation on the motif CTAG is involved in influencing virulence and pathogenicity, or *Clostridioides difficile*, where methylation at the motif CAAAAAA regulates sporulation [33]. Pathogenic bacteria employ DNA methylation as a versatile strategy for phase variation, allowing them to adapt to environmental changes and evade host immune responses [34]. According to an analysis of phase-variable expression of restriction–modification systems (type I and type III) in pathogenic bacteria adapted to humans, multiple gene expressions were found to be controlled by methylation modifications [35]. Phasevarion is the name given to these structures (phase-variable regulons) [35]. The evasion of the host immune response is facilitated by phase variation of switching between virulence and avirulence phenotypes [36,37]. In the monoderm pathogen *Streptococcus pneumoniae*, type I phasevarions have been shown to regulate virulence through global epigenetic changes [38]. The different strains locked into one epigenetic state (by expressing only one out of six possible specificity subunit genetic variants) varied in colony opacity and morphology and this was also correlated with virulence [39]. Phase-variable genes, responsible for surface structures like pili or outer membrane proteins, undergo reversible and stochastic changes in expression. This was observed in a uropathogenic *E. coli* strain, responsible for urinary tract infections [40]. This strain expresses various pathogenicity factors (e.g., biofilm formation, type I pili, pyelonephritis-associated pilus P (Pap), and the siderophore aerobactin). Phase-variation mechanisms induce changes in the DNA methylation patterns, and as a consequence, the expression of Pap pili is regulated. This regulation is driven both by DNA methylation and external stimuli (mainly temperature and glucose concentration) [40]. The transcriptional control element Papl binds to a specific non-methylated regulatory DNA region containing the sequence GATC and facilitates the formation of an active transcriptional complex [41]. DNA methylation patterns dictate the transcriptional status of specific genes within promoter regions, leading to the rapid alteration of surface structures and facilitating immune evasion. This dynamic interplay highlights the adaptability conferred by DNA methylation in the arms race between pathogens and their hosts.

DNA methylation also contributes to the regulation of genes involved in quorum sensing, a communication system that allows bacterial populations to coordinate behavior in a density-dependent manner. Methylated DNA motifs function as key elements in the hierarchical control of quorum-sensing circuits. DNA methylation patterns influence the binding affinity of transcriptional regulators, orchestrating the synchronized expression of genes involved in social behaviors such as biofilm formation, virulence, and antibiotic resistance. DNA methylation has been demonstrated to play an important role in regulating the expression of *Burkholderia cenocepacia* genes involved in biofilm formation, cell aggregation, and motility [2,42]. Furthermore, DNA methylation influences quorum-sensing-related virulence in pathogenic strains of *E. coli*, *Salmonella*, *Vibrio*, *Yersinia*, *Haemophilus*, and *Brucella*. For instance, genes encoding antibiotic-modifying enzymes in an antibiotic susceptible *Mycobacterium abscessus* strain have higher rates of methylation modification compared to the antibiotic resistance strain, which may indicate differences in the regulation of their expression [43]. DNA adenine methylase (Dam) methylation regulates other quorum-sensing-related phenotypes, such as those related to the invasion of epithelial cells in *Salmonella enterica* [44] and *Haemophilus influenzae* [45], the secretion of *Yersinia* outer membrane proteins [46], and the synthesis of Std fimbriae in *Salmonella* [47].

Bacterial DNA MTases can also directly affect host cells in host–microbe interactions. Indeed, the epigenetic landscape of the host can be modified by secreted nucleomodulins, bacterial effectors that target the nucleus of infected cells. A subclass of such molecules encodes DNA MTase activities, targeting both host DNA and histone proteins, leading to important transcriptional changes in the host cell [48]. In pathogens where DNA methylation is involved in the virulence process, drugs inhibiting such enzymes can be expected to attenuate virulence by transforming wild-type bacteria into phenocopies of DNA methyltransferase mutants. In principle, such drugs should be harmless to the host because N6-methyladenine is rare, if not absent, in mammalian cells [49]. The advantage of such drugs may be the absence of lethal effects, with a reduced frequency of insurgence of resistant mutants [50]. Another effect of DNA methyltransferase antagonists concerns the Dam inhibitors, which are able to enhance the efficacy of antibiotics [51]. The exposure to beta-lactams causes oxidative damage, which induces the SOS response including error-prone DNA repair and consequently mismatch production. In the absence of DNA strand discrimination, for example when the DNA methylation process is altered, the MutHLS MMR system generates double-strand breaks. Such breaks further activate the SOS response, thus generating a toxic feedback loop that potentiates the lethal action of the antibiotic [51]. Moreover, in the field of phage therapy, DNA methylation can increase the effect of the treatment thanks to the insertion of genes encoding DNA MTases capable of slowing down the recognition of the phage as a non-self-element by host restriction–modification systems [52]. This unique interplay between DNA methylation and developmental processes challenges traditional notions of prokaryotic simplicity. The reversible nature of DNA methylation allows for the rapid adaptation of bacterial populations to changing environmental conditions, leading to the emergence of subpopulations with advantageous traits. Understanding the interplay between epigenetic maintenance mechanisms and gene regulatory networks can provide novel information and models on the significance of epigenetic inheritance in prokaryotic life.

## 5. Epigenomics and Genome Evolution

The presence of DNA methylation offers a hint into the adaptive strategies and evolutionary forces that shape microbial life. Ancient lineages of bacteria and archaea bear witness to the presence of DNA methyltransferases, highlighting the deep-rooted nature of this epigenetic modification. The conservation of DNA methylation across diverse prokaryotic taxa stresses its functional significance in microbial evolution. Phylogenetic analyses reveal both conserved and lineage-specific aspects of prokaryotic DNA methylation [2]. Certain methylation motifs are shared among distantly related taxa, indicating ancient origins and possible functional conservation. Simultaneously, lineage-specific innovations in methylation patterns highlight the ongoing evolutionary experimentation that drives microbial diversification. The evolutionary dynamics of DNA methylation in prokaryotes are linked to adaptive functions (briefly summarized in the previous paragraph) and the exchange of genetic material [53]. Unlike the relatively uniform CpG dinucleotide methylation in eukaryotes, prokaryotic methylation motifs exhibit remarkable variability. Species-specific and even strain-specific motifs abound, reflecting the intricate relationship between methylation and genomic context [2,54]. The distribution of DNA methylation across prokaryotic taxa showcases a mosaic of patterns that defy a one-size-fits-all model. While some species exhibit extensive methylation throughout their genomes, others confine methylation to specific genomic regions or regulatory elements. Understanding this variability is crucial for deciphering the functional implications of methylation in different microbial lineages. Finally, the plasticity of prokaryotic genomes extends to their epigenomes, with environmental factors shaping methylation patterns in response to changing conditions, thus reflecting the intricate interplay between epigenetic regulation and the selective pressures exerted by the environment [55]. Indeed, the diversity of methylation patterns in prokaryotes is intimately linked to ecological niche specialization. Different microbial lineages, each adapted to specific environmental niches, exhibit unique methylation signatures that contribute to their ecological success [56]. If and how much DNA methylation in prokaryotes serves as a form of epigenetic memory, enabling microbial populations to retain information about past encounters with environmental challenges, has still to be defined. This memory could contribute to adaptive responses, allowing prokaryotes to swiftly adjust their gene expression profiles in anticipation of recurring stressors [57]. Other than adaptation, DNA methylation patterns are powerful agents of population differentiation and speciation when related to R-M systems, since they can strongly influence part of the gene flow (e.g., transduction) [58]. Since R-M systems are barriers to the acquisition of genetic elements through horizontal transfer [1,3], different lineages can arise in bacterial populations due to the presence of novel R-M systems. Additionally, R-M systems can rarely be harbored on mobile genetic elements (MGEs) [59], thus impacting recursively on gene flow after the movement of the MGEs. A recent study on a strain of *Paenibacillus polymyxa* showed the presence of a type I R-M system in an MGE carried by this strain and an average frequency of the DNA methylation motifs in MGEs lower than that in the genome, suggesting a role of this type I R-M system in restricting MGEs during the genomic evolution of *P. polymyxa* [60]. In the plant symbiotic nitrogen-fixing bacterium *Sinorhizobium meliloti*, the *hsdR* gene, encoding a restriction system, was shown to strongly impact gene flow between *S. meliloti* and the sister species *Sinorhizobium medicae*, possibly indicating a role in sympatric speciation, since these two species can be found in the same environment (the plant rhizosphere). More recently, still concerning the *Sinorhizobium* genus, different species and also different strains of the same species *S. meliloti* were shown to harbor some different methylated DNA motifs [15], supporting the hypothesis of a link between genome evolution driven by horizontal gene transfer and recombination. R-M systems could be relevant to the explanation of the high genome diversity found in nature, especially in species with a large open pangenome, such as *S. meliloti*. Moreover, other studies have shown conspicuous differences in R-M systems in *Bacillus velezensis* [61] and in *Mycoplasma agalactiae*, with the presence of strain-specific motifs and orphan DNA MTases affecting the rate of horizontal gene transfer events among *M. agalactiae* strains [62].

## 6. Computational Tools for Bacterial Epigenomic Studies

**Figure 2:**
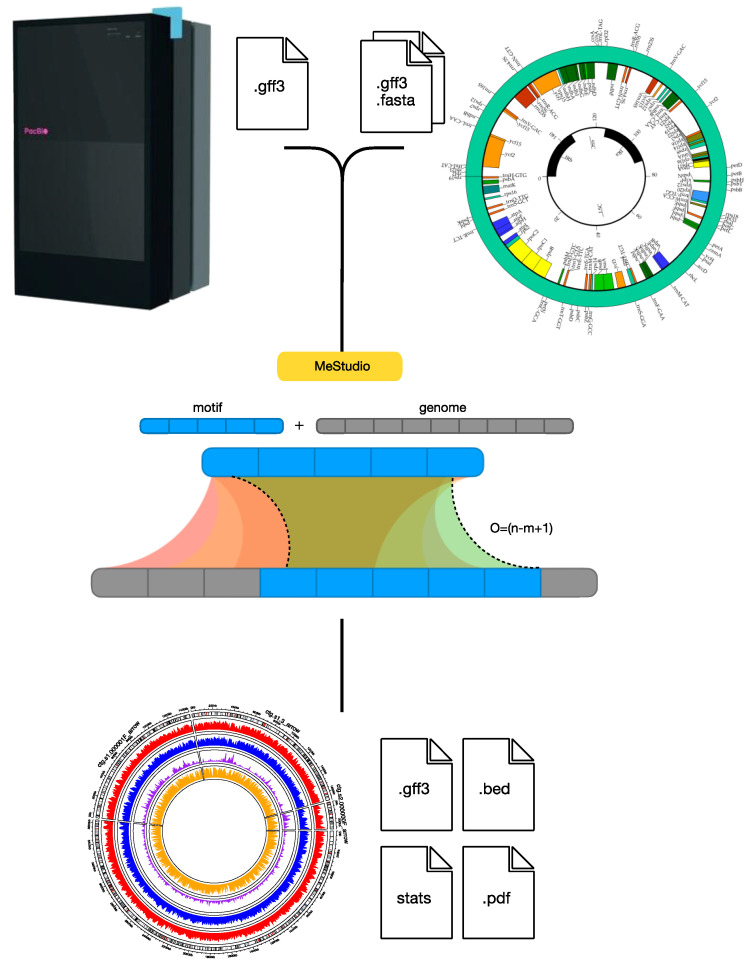
An analysis of genome-wide DNA methylation data. Here, the MeStudio [68] workflow is shown. Starting from genome sequencing obtained by the SMRT sequencing technique and genome annotation file, the recurrent methylated DNA motifs identified are searched within the genome sequence(s). Three main outputs are obtained, a .gff3 file and .bed file with annotated methylated sequences and their occurrence with respect to genomic regions (e.g., coding sequences, promoters, etc.) and a file with statistics of the occurrence of motifs and the percentage of their methylation. Visual outputs shown as circus plots may allow us to easily identify regions with peculiar methylation patterns and are saved as PDF files.

As outlined in the previous paragraphs, the interest in analyses of genome-wide methylation profiles is gaining momentum, as evidence for multiple roles of methylated DNA bases is accumulating. Moreover, single-molecule sequencing technologies, such as Pacific Biosciences single-molecule real-time (SMRT) sequencing [63] and Nanopore ONT sequencing [64], allow us to easily identify methylated bases at a low cost. As the price decreases and accessibility to such sequencing apparatuses increases, the number of available data are becoming important. However, now, we need easy-to-use and powerful computational tools. While several instruments have already been developed for the analysis of DNA methylation profiles deriving from bisulfite sequencing and microarrays [65], there are only a few packages for Nanopore ONT and SMRT methylation data [66,67]. Tools have also been developed to analyze DNA methylation profiles on metagenomic data (https://github.com/hoonjeseong/Meta-epigenomics (accessed on 15 March 2024), but all of these computational packages do not offer the possibility of mapping methylated motifs with respect to annotated functional features of the genomes (e.g., coding sequences, promoters, or other relevant features or genomic positions). Our laboratory recently developed a pipeline (MeStudio [68]) for extracting DNA methylation information from SMRT sequencing data, allowing for the comparison of the position of methylated motifs with respect to the genome annotation. Report outputs can be used to compare the methylation status of DNA regions in different environmental contexts or to perform genome-wide comparisons of strains. MeStudio consists of a pipeline and uses a string-matching algorithm to map motif sequences to the reference genome (Figure 2). The required input data consist of the FASTA file containing the genome sequence, the genomic annotation file, and the GFF3 file containing the methylated nucleotide positions from SMRT Link software (https://www.pacb.com/smrt-link/) of Pacific Biosciences DNA sequencers. Outputs are datasets of methylated motifs with respect to genome positions, including the presence of coding sequences or other relevant annotation features listed in the GFF3 file. Such outputs can be used to statistically infer the uneven distribution of methylation and possibly produce different types of graphical representations. However, even this tool needs improvement, since we can measure the methylated bases not included in motifs only indirectly and the input from Nanopore ONT sequencing cannot be processed at the moment.

## 7. Conclusions and Future Directions

In the intricate landscape of prokaryotic epigenetics, the detection and characterization of DNA methylation still present several challenges. Prokaryotic DNA methylation patterns are mainly studied and identified in relation to motifs, that is, recurrent sequences of bases where DNA MTases act. However, there can be also methylated bases where a well-defined consensus recognition motif is missing. To extract DNA methylation information from sequencing data and directly quantify and compare the position of methylated sites with respect to genome-derived features (e.g., coding and non-coding regions), bioinformatic pipelines are needed. The position of methylated sites throughout the genome is of key importance when the interest is in understanding the role that epigenetic modifications have in gene expression control and phenotypic plasticity [68]. The integration of computational methods with experimental approaches is vital for predicting methylation motifs in prokaryotic genomes. Machine learning algorithms and bioinformatics tools aid in the identification of potential methylation motifs, guiding the design of targeted experiments [69]. The diversity of prokaryotic species, each harboring unique methylation patterns, poses a challenge in establishing a comprehensive functional understanding across taxa. Moreover, genes regulated by methylation can vary widely between species and even within different strains of the same species [62]. The lack of a “universal code” governing the effects of methylation on gene expression complicates the interpretation of observed methylation patterns. Prokaryotic DNA methylation has been implicated in influencing various phenotypic traits, including the previously mentioned antibiotic resistance, stress response, and virulence. However, the specific mechanisms through which methylation contributes to these traits remain elusive in many cases. Unraveling the functional significance of methylation events is crucial for deciphering the adaptive strategies employed by prokaryotes in response to environmental challenges.

### The Need for Experimental Validation of Predicted Effects

Predicting the functional consequences of DNA methylation in bacteria mainly relies on in silico analyses and correlation studies. However, to establish causation and truly understand the impact of methylation on gene expression and cellular processes, rigorous experimental validation is required. One avenue for experimental validation involves targeted mutagenesis studies, where specific DNA MTases or demethylases are selectively disrupted [70,71] and the downstream effects on gene expression and phenotypic traits can be observed. This approach, however, requires a meticulous understanding of the target genes and their regulatory elements. Paradigmatic examples of this approach are presented by the studies on cell cycle-related methylation patterns, such as those already mentioned for *C. crescentus* and *E. coli* [4,19]. Integrating transcriptomic and proteomic analyses into methylation studies provides a holistic view of the functional consequences of DNA methylation. High-throughput sequencing technologies enable the simultaneous profiling of gene expression and methylation patterns, facilitating the identification of direct correlations between epigenetic modifications and changes in the cellular phenotype [29,72], and providing insights into the functional constraints and innovations that shape microbial genomes [29,72]. Finally, comparative epigenomics can offer valuable insights for the functional interpretation of DNA methylation patterns and their relevance to prokaryotic evolution. Studying methylation patterns in closely related species or strains with distinct phenotypic traits can reveal the selective pressures that govern the retention or loss of specific methylation events during evolution [73]. Finally, greater efforts should be made to move epigenomics into the context of ecological genetics, either through population epigenomic analyses, experimental evolution studies, or environmental meta-epigenomics. Experimental evolution, leveraging controlled laboratory conditions, can offer a powerful tool to investigate the adaptive trajectories of DNA methylation [69,74,75]. Tracking the dynamics of methylation patterns over multiple generations in response to specific selective pressures can provide experimental validation of the functional significance attributed to observed methylation events and decipher the presence and relevance of transgenerational inheritance in prokaryotes. Environmental meta-epigenomics could enable the exploration of DNA methylation in microbial communities and its possible ecological roles on ecosystems’ dynamics [56], but could also help in identifying signatures of genomes for performing higher-quality productions of MAGs (metagenome-assembled genomes [56,76]).

## Data Availability

No new data were created or analyzed in this study. Data sharing is not applicable to this article.
